# Salivary Excretion Of Torque Teno Virus And Sars-Cov-2 In Children Who Underwent Kidney Transplantation

**DOI:** 10.1590/0034-7167-2025-0285

**Published:** 2026-09-18

**Authors:** Ísis Oliveira Arruda, Richarlisson Borges de Morais, Rodrigo Melim Zerbinati, Karen Renata Nakamura Hiraki, Maria Cristina de Andrade, Dulce Aparecida Barbosa, Paulo Henrique Braz-Silva, Monica Taminato

**Affiliations:** I Universidade Federal de São Paulo. São Paulo, São Paulo, Brazil; II Universidade Federal de Uberlândia. Uberlândia, Minas Gerais, Brazil; III Universidade de São Paulo. São Paulo, São Paulo, Brazil

**Keywords:** Torque Teno Virus, COVID-19, SARS-CoV-2, Kidney Transplant, Pediatrics., Torque Teno Virus, COVID-19, SARS-CoV-2, Trasplante renal, Pediatría.

## Abstract

**Objectives:**

to verify the salivary excretion of Torque Teno Virus (TTV) in children and adolescents who underwent kidney transplantation and to correlate the number of viral copies with the polymerase chain reaction test for SARS-CoV-2.

**Methods:**

a cross-sectional observational study conducted at the post-kidney transplant outpatient clinic of *Hospital do Rim e Hipertensão*.

**Results:**

thirty-one patients were included, with a predominance of males (54.8%) and ages between 13 and 15 years (38.7%). Salivary excretion of TTV was detected in 96.7% of participants, of whom 36.7% also tested positive for SARS-CoV-2. However, no significant association was found between the two viruses. A moderate negative correlation (-0.463) was observed between TTV levels and serum tacrolimus concentration.

**Conclusions:**

salivary excretion of TTV was identified in children and adolescents who underwent kidney transplantation. Although no correlation was observed between TTV and SARS-CoV-2, an association was found between TTV and serum tacrolimus levels.

## RESUMEN

**Objetivos:**

verificar la excreción salival de Torque Teno Virus (TTV) en niños y adolescentes con trasplante renal y correlacionar el número de réplicas virales con la prueba de reacción en cadena de la polimerasa para SARS-CoV-2.

**Métodos:**

estudio observacional transversal, realizado en el ambulatorio de seguimiento pos-trasplante renal del *Hospital do Rim e Hipertensão*.

**Resultados:**

se incluyeron 31 pacientes, con predominio del sexo masculino (54,8%) y edades entre 13 y 15 años (38,7%). La excreción salival de TTV se detectó en el 96,7% de los participantes, de los cuales el 36,7% también resultaron positivos para SARS-CoV-2. Sin embargo, no se encontró una asociación significativa entre ambos virus. Se observó una correlación moderada y negativa (-0,463) entre los niveles de TTV y la concentración sérica de tacrolimus.

**Conclusiones:**

la excreción salival de TTV fue identificada en niños y adolescentes con trasplante renal. Aunque no se observó correlación entre TTV y SARS-CoV-2, se encontró asociación entre TTV y los niveles séricos de tacrolimus.

## INTRODUCTION

Torque Teno Virus (TTV), an anellovirus, was first found in 1997 in the blood plasma of Japanese people who developed hepatitis after transfusion^(1-4)^. TTV exhibits genetic diversity of more than 29 species, with approximately 95% predominating in the world population^(2,4)^, regardless of age, economic status, and health conditions^(5-7)^.

TTV is considered an orphan/commensal virus, as the scientific community has not yet been able to identify its association with any disease^(8-10)^, being considered part of the human virome in healthy people. Some studies report that primary infection with the virus occurs in childhood, leading to chronic infection and subsequent stable viremia^(11)^, but its mode of replication and transmission is still unknown^(12)^.

In recent years, molecular biology techniques have revealed that TTV constitutes more than 65% of the virome in post-transplant patients and only 10% in immunocompetent individuals^(4,13,14)^. Plasma levels of TTV DNA showed fluctuations in immunosuppressed patients, meaning that high TTV viral loads are directly associated with the intensity of an individual’s immune function^(15-18)^. Thus, immune disorders, anti-inflammatory or immunosuppressive medications can affect the immune balance and, consequently, favor TTV replication^(8,11,19)^. This allows for the identification of high levels of TTV viral load and its detection in different samples, such as plasma, saliva, and serum^(8,17)^.

In this context, considering that Coronavirus Disease 2019 (COVID-19) has been directly related to inflammatory and immune dysregulation, these alterations are associated with the severity of the clinical picture of COVID-19^(20)^. Therefore, it became necessary to understand the interaction of SARS-CoV-2 with other pathogens and the influence of these interactions and other underlying clinical conditions on the clinical picture of the disease.

Kidney transplant recipients have a high risk of severe COVID-19 due to their immunosuppressed state^(21)^. Furthermore, studies show that immunosuppressed patients, including kidney transplant recipients, have greater TTV dispersion when compared to their healthy counterparts^(22)^. Considering this increased risk associated with SARS-CoV-2 co-infection, there are reports in the literature indicating changes in the viral excretion profile for TTV^(21)^. Given the above, it is highlighted that renal transplant patients have a double association of factors that alter their immunological status: chronic kidney disease (CKD) and the use of immunosuppressive drugs^(23)^. These clinical and epidemiological characteristics motivated the present study in order to fill the existing gap in the literature regarding the association between the number of viral replicates and TTV and SARS-CoV-2 co-infection.

## OBJECTIVES

To verify the salivary excretion of TTV in children and adolescents who underwent kidney transplantation and to correlate the number of viral copies with the polymerase chain reaction (PCR) for SARS-CoV-2.

## METHODS

### Ethical aspects

The study was conducted in accordance with national and international ethical guidelines. It was approved by the Research Ethics Committee of *Universidade Federal de São Paulo* (UNIFESP), whose opinion is attached to this submission.

### Study design

This is a cross-sectional observational study, in accordance with STrengthening the Reporting of Observational studies in Epidemiology recommendations^(14)^ for observational studies in epidemiology^(24)^.

### Study period and location

The study was conducted in the post-renal transplant outpatient clinic of *Hospital do Rim e Hipertensão* (HRim), *Fundação Oswaldo Ramos*, and UNIFESP.

Biological material (saliva) and sociodemographic and clinical data from electronic medical records were collected over a 30-day period, from January 27 to February 26, 2022, from all children and adolescents who were undergoing post-renal transplant outpatient follow-up at the participating institution.

### Study population

This is a convenience sample, composed of 31 individuals aged 0 to 18 years, with end-stage CKD, under outpatient follow-up at HRim, who underwent kidney transplantation.

### Inclusion and exclusion criteria

The study included pediatric patients aged 18 years or younger who had undergone kidney transplantation within the 12 months prior to the data collection period and were being followed up at the HRim post-transplant outpatient clinic.

### Study protocol

Parents or guardians of individuals eligible for the research were approached in the waiting room. After explaining and understanding the objectives research and all stages of the, they were invited to participate and sign the Informed Consent Form (ICF). With the ICF signed, the study objectives and the procedures for collecting the saliva sample were explained to participants (child and/or adolescent) in appropriate language before obtaining their assent to participate. A saliva sample was collected from the participant undergoing renal replacement therapy (RRT). Data related to sociodemographic characteristics, history, and clinical evolution were obtained from medical records using a semi-structured instrument developed by the researchers based on a literature review. It is important to note that the study did not generate any changes in their care routine or alterations in treatment.

Saliva samples were obtained using the standardized method of the commercial device Salivette^®^ (Sarstedt, Germany). The tubes were identified and stored in a refrigerator (between 2°C and 8°C) until the end of the scheduled collections for the shift. Subsequently, the samples were centrifuged at 1,000 x g for two minutes at a temperature of 20°C, then aliquoted and frozen in a freezer at -80°C.

Nucleic acid (DNA/RNA) was extracted from 200 µl of each saliva sample using the Extracta kit - Viral RNA and DNA (*Loccus do Brasil*, Ltda.) in the DNA and RNA Extractor and Purifier - EXTRACTA 32. To detect SARS-CoV-2 viral RNA, molecular analysis was performed using the SARS-CoV-2 RT-qPCR Reagent kit (PerkinElmer, Finland), following the manufacturer’s instructions. For the quantification of TTV viral replicas, quantitative PCR was performed using the QuantStudio™ 6 Flex Real-Time PCR System (Applied Biosystems^®^ - Thermo Fisher, USA) in 45 amplification cycles, as previously described in the literature^(25)^.

### Statistical analysis

Data analysis was performed using descriptive statistics, with measures of frequency, central tendency (mean and median), and dispersion (standard deviation). In addition, comparative analyses were conducted between groups according to the nature of the variables.

When comparing numerical variables, the non-parametric Mann-Whitney test was used, and for categorical variables, Fisher’s exact test was used. To correlate the number of TTV replicates with the other variables, Pearson’s correlation was used when the variables had a normal distribution, and Spearman’s correlation was used for those that did not have a normal distribution. The significance level adopted was 0.05, equivalent to a 95% confidence level.

## RESULTS

Thirty-one children and adolescents who had undergone kidney transplantation and met the eligibility criteria for this study were included. As for participant sociodemographic data, the prevalence of males (17/54.8%), the 13-15 year age range (12/38.7%), mixed race (16/51.6%), incomplete primary education (18/58.1%), family income of up to one minimum wage (19/61.3%), and non-beneficiaries of the Brazilian National Social Security Institute (*Instituto Nacional do Seguro Social*) (18/58.1%) stood out. Regarding transportation (19/61.3%), they reported using a car to go to the post-kidney transplant outpatient clinic, with an average travel time exceeding three hours (17/54.8%) (Table 1).

**Table 1:** Descriptive analysis of sociodemographic, clinical, and laboratory characteristics of children and adolescents who underwent kidney transplantation, São Paulo, São Paulo, Brazil, 2024 (N=31)

CHARACTERISTICS	n (%)
**Sex**	
Male	17 (54.8%)
Female	14 (45.2%)
**Age**	
5 to 8 years old	4 (12.9%)
9 to 12 years old	7 (22.6%)
13 to 15 years old	12 (38.7%)
16 to 18 years old	8 (25.8%)
**Skin color/ethnicity**	
White	8 (25.8%)
Indigenous	1 (3.2%)
Black	6 (19.4%)
Mixed-race	16 (51.6%)
**Education**	
Preschool	1 (3.2%)
Incomplete elementary school	18 (58.1%)
Incomplete high school	11 (35.5%)
Does not attend regular school	1 (3.2%)
**Family income (minimum wages)**	
Up to 1	19 (61.3%)
1-3	11 (35.5%)
**SOCIODEMOGRAPHIC DATA**	
**Receives INSS benefits**	
Yes	13 (41.9%)
No	18 (58.1%)
**Means of transport used to go to the post-transplant clinic**	
Car	19 (61.3%)
Bus	12 (38.7%)
Train	3 (9.7%)
Subway	9 (29%
Airplane	4 (12.9%)
**Travel time (home - post-transplant clinic)**	
Up to one hour	8 (25.8%)
From one to two hours	3 (9.7%)
From two to three hours	3 (9.7%)
More than three hours	17 (54.8%)
**Residence situation**	
Owned	17 (54.8%)
Rented	8 (25.8%)
Given	6 (19.4%)
**Residence characteristic**	
Masonry	31 (100%)
**Number of rooms in the residence**	
0	1 (3.2%)
2	3 (9.7%)
3	2 (6.5%)
4	10 (32.3%)
5	10 (32.3%)
6	4 (12.9%)
9	1 (3.2%)
**Number of residents in the same household**	
1	4 (12.9%)
3	9 (29%)
4	8 (25.8%)
5	5 (16.1%)
6	4 (12.9%)
8	1 (3.2%)
**Piped water**	
Yes	28 (90.3%)
No	3 (9.7%)
**Sewer system**	
Yes	26 (83.9%)
No	5 (16.1%)
**CLINICAL DATA**	
**CKD etiology**	
Undetermined	9 (29%)
Hemophagocytic syndrome	1 (3.2%)
Nephrotic syndrome	6 (19.4%)
Secondary to acute kidney injury	1 (3.2%)
Congenital anomalies of the kidney and urinary tract	6 (19.4%)
Immunoglobulin A nephropathy	1(3.2%)
Neurogenic bladder	2 (6.5%)
Renal dysplasia	1 (3.2%)
Polycystic kidney disease	1 (3.2%)
Bilateral vesicoureteral reflux	3 (9.7%)
Arnold-Chiari type II	1 (3.2%)
Hemolytic-uremic syndrome	1 (3.2%)
Autosomal recessive polycystic kidney disease	1 (3.2%)
Nephropathic cystinosis	1 (3.2%)
**There was previous RRT**	
Yes	28 (90.3%)
No	3(9.7%)
**Which previous RRT**	
Hemodialysis	27 (87.1%)
DPA	1 (3.2%)
**Blood type**	
A	10 (32.3%)
B	2 (6.5%)
AB	1 (3.2%)
O	18 (58%)
**HR factor**	
Positive	28 (90.3)
Negative	3 (9.7%)
**Received a blood transfusion**	
Yes	16 (51.6%)
No	15 (48.4%)
**Number of transfusions in the last 12 months**	
<5	15 (48.3%)
6 to 10	1 (3.2%)
**Donor type**	
Deceased	30 (96.7%)
Alive kinship	1(3.3%)
**Influenza-vaccinated (2022)**	
Yes	17 (54.8%)
No	14 (45.2%)
**COVID-19-vaccinated**	
Yes	29 (93.5%)
No	2 (6.5%)
**LABORATORY TESTS**	
**Post-transplant urine culture**	
Positive	1 (3.2%)
Negative	19 (61.3%)
**Isolated germ in urine culture**	
*Klebsiella pneumoniae*	1 (3.2%)
**Post-transplant blood culture**	
Positive	0 (0%)
Negative	18 (58%)

*N - absolute number; % - relative number; INSS - Brazilian National Institute of Social Security (Instituto Nacional do Seguro Social); CKD - chronic kidney disease; RRT - renal replacement therapy; HD - hemodialysis; TX - kidney transplant.*

Concerning residential characteristics, most own their own home (17/54.8%), built of masonry (31/100%), with four or five rooms (10/32.3%), and most have three residents in the same dwelling (9/29%). Most have piped water (28/90.3%) and a sewage system (83.9%) (Table 1).

In relation to clinical data, CKD etiology (9/29%) was considered undetermined, with nephrotic syndrome and congenital anomalies of the kidney and urinary tract being the most prevalent (6/19.4%). It is noteworthy that 28 (90.3%) underwent another method of RRT before kidney transplantation, and 27 (87.1%) underwent hemodialysis. The most prevalent blood type was “O” (18/90.3%), with a predominance of the Rh-positive factor (28/90.3%). Patients received transfusions on fewer than five occasions (15/48.3%), underwent deceased donor transplantation (30/96.7%), and received influenza (17/54.8%) and coronavirus disease 2019 vaccines (29/93.5%). As for laboratory tests, one patient had a positive urine culture (1/3.2%) for *Klebsiella pneumoniae*, and all blood cultures collected were negative.

**Figure 1:**
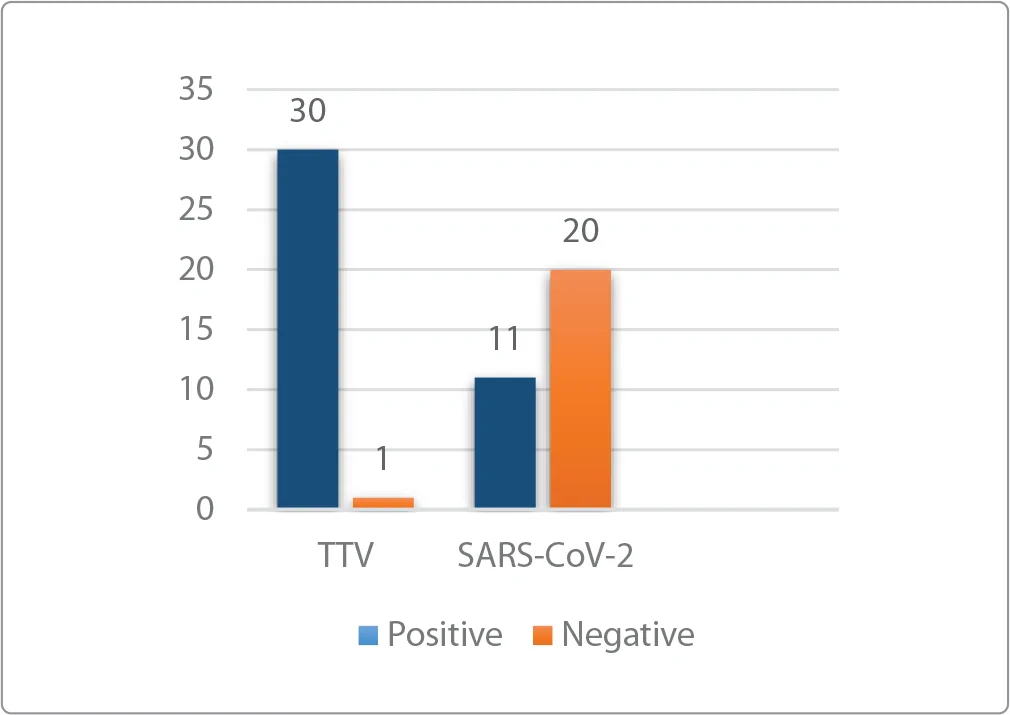
Result of the qualitative polymerase chain reaction test for Torque Teno Virus and SARS-CoV-2 in children and adolescents who underwent kidney transplantation, São Paulo, São Paulo, Brazil, 2024 (N=31)

According to Figure 1, among the 31 study participants, 30 (96.8%) showed dispersed TTV viral replicas, while in one (3.2%) no such replicas were found in the saliva. Furthermore, 11 participants (35.5%) tested positive for salivary PCR concomitantly with TTV. It is noteworthy that, when applying Fisher’s exact test, there was no statistically significant association between TTV and SARS-CoV-2 viruses (p=1).

When comparing the sociodemographic, clinical, and laboratory variables of the kidney transplant children and adolescents included in this study with the qualitative PCR for TTV, no statistically significant association was found with any of the variables studied.

When comparing the number of TTV viral replications identified in the saliva of children and adolescents who had undergone kidney transplants with the qualitative result of salivary PCR for SARS-CoV-2, no relationship was observed between having or not having the disease and the number of TTV viral replications (p=0.426) (Table 2).

**Table 2:** Comparison between the number of viral replications of Torque Teno Virus and quantitative real-time reverse transcription polymerase chain reaction for SARS-CoV-2 in children and adolescents who underwent kidney transplantation, São Paulo, São Paulo, Brazil, 2024 (N=31)

	RT-PCR SARS-CoV- 2		
**TTV log_10_ CP/mL**	**Positive**	**Negative**	*p* value^^*^^
**Mean (±)**	9.304 (1.656)	8.768 (1.925)	0.426

TTV - Torque Teno Virus; RT-PCR - quantitative real-time reverse transcription polymerase chain reaction; log10 - base-10 logarithm; CP/mL - copies per milliliter;

* Mann-Whitney test.

Table 3 shows the correlation between the number of viral replicates of TTV (log_10_ CP/mL) and clinical variables. It can be highlighted that there is a moderate negative correlation between serum tacrolimus and TTV (log_10_ CP/mL). For the other quantitative variables, the correlation with TTV log_10_ CP/mL was weak.

**Table 3:** Correlation between Torque Teno Virus and sociodemographic, clinical, and laboratory variables in children and adolescents who underwent kidney transplantation, São Paulo, São Paulo, Brazil, 2024 (N=31)

Correlation	TTV log_10_ CP/mL
Travel time (hours)	-0,129^*^
Number of rooms	0,181^*^
Residents in the same house	-0,083^*^
Age	0,034^*^
Weight	0,105^**^
Height	0,157^*^
RRT start time	0,042 ^*^
TX time (years)	-0,294^**^
Treatment time (months)	-0,294^**^
Number of post-transplant UTI episodes	0,237 ^*^
Number of post-transplant BSI episodes	0,308^*^
Pre-transplant glucose	-0,065^**^
Pre-transplant creatinine	0,298^**^
Ischemia time (in hours)	-0,369^*^
Cold ischemia time (in hours)	-0,365^*^
Warm ischemia time (in minutes)	0,267^*^
Serum tacrolimus	-0,463^**^
Cr	-0,179^*^
K	-0,171^*^
Ht	-0,167^**^
Hb	-0,149^**^
Leu	-0,197^**^

RRT - renal replacement therapy; TX - kidney transplant; UTI - urinary tract infection; BSI - bloodstream infection; Cr - creatinine; K - potassium; Ht - hematocrit; Hb - hemoglobin; Leu - leukocytes; log10 - base-10 logarithm; CP/mL - copies per milliliter;

* Spearman’s correlation;

** Pearson’s correlation.

## DISCUSSION

This study investigated salivary TTV excretion in children and adolescents undergoing kidney transplantation, assessing the association between the number of TTV viral replications and SARS-CoV-2 detection. Results revealed that 96.8% of participants tested positive for TTV, with a mean viral load of 9.304 log_10_. In individuals co-infected with SARS-CoV-2, there was a slight variation in TTV viral load (8.768 log_10_), without statistically significant difference.

Participant sociodemographic distribution was consistent with previous studies, which indicate a higher prevalence of TTV in males^(23,26)^. A study in Italian blood donors demonstrated a prevalence of 65%, with a progressive increase in virus detection in individuals under 60 years of age, reaching 45% between 18 and 22 years of age^(27)^. Another study conducted with 313 healthy individuals reported that 70% of plasma samples positive for TTV were from individuals between 20 and 30 years old, with 78% in the 50-60 age group and 80% in the 80-year-old age group. Furthermore, in healthy participants, there was an association between sex and viral load, with lower levels in young women compared to men in the same age group^(28)^.

As for the interaction between TTV and COVID-19, a study conducted by the “Corona São Caetano Program” in the municipality of São Caetano do Sul, Brazil, revealed the presence of TTV in 54.6% of individuals who tested positive for SARS-CoV-2 and in 62.7% of controls. The study also identified a significant reduction in the viral load of TTV after ten days of clinical evolution of COVID-19, while in negative individuals, the load remained unchanged^(29)^. Similarly, a case report involving a kidney transplant patient demonstrated an increase in TTV viral load during SARS-CoV-2 infection (7.14 log_10_/7.87 log_10_). Comparing this finding with the number of TTV viral replications at one and six months post-transplant (5.6 log_10_ and 5.9 log_10_, respectively), it is noted that there was an increase in TTV viral shedding during SARS-CoV-2 infection, suggesting its potential usefulness as a biomarker of immunosuppression^(30)^.

Studies indicate that monitoring TTV viral load can be a reliable indicator of immune status in transplant patients^(31)^. Evidence demonstrates the relationship between variations in TTV levels and the immune function of transplant patients^(32-34)^.

The use of immunosuppressive drugs reduces the risk of transplant rejection, but increases the risk of infection^(35)^. Studies show that quantifying the viral load of TTV has many benefits, since PCR can be done in a few hours, is available in many health institutions, and is an already established technique. Furthermore, the viral load of TTV can predict rejection^(34,35)^ and infection in transplant patients, which contributes to monitoring and clinical decision-making^(36)^.

Prospective studies suggest that quantifying TTV viral load 180 days after kidney transplantation may aid in calibrating immunosuppressive therapy, reducing the risks of infection and rejection^(37,38)^. Researchers propose an ideal viral load range between 4.6 and 6.6 log_10_ CP/mL 90 days post-transplant. This result indicated that values below 4.6 log_10_ may be associated with rejection, and values above 6.6 log_10_ may increase the risk of infection^(39)^.

Another Austrian study with 386 transplanted adults observed that 68 participants rejected the transplant and 207 presented 472 episodes of infection in the first 12 months. After three months post-transplant, the study reported that the cutoff levels for the TTV range for rejection were the median TTV (3.5 × 10^6^ c/mL) and the median for the occurrence of infection (3.9 × 10^8^ c/mL)^(40)^.

With the emergence of the COVID-19 public health emergency, many studies have proposed linking SARS-CoV-2 infection to different clinical situations, including co-infection with other pathogens. One study compared COVID-19 mortality at a kidney transplant center from March 2020 to December 2022 to the previous five years (2014-2019). It identified that before the pandemic, 1,025 lives were lost per year, while during the pandemic there were 1,429 deaths, with COVID-19 responsible for 59% of those deaths^(20)^. COVID-19 immunization in patients with CKD is a crucial aspect to consider. Studies emphasize the safety and efficacy of vaccines in children with comorbidities, reinforcing that the benefits outweigh the risks^(41)^.

A retrospective cohort study conducted in Rome with 172 solid organ transplant recipients (kidney and lung) and 72 blood donors, cross-matched with the healthy population, assessed the relationship between TTV viral load and humoral response after administration of doses of the messenger RNA vaccine against COVID-19, BNT162B2. It was found that 52% of patients had antibodies after the second dose of the vaccine, 76% after the third dose, and 87% after the fourth dose. Concerning the quantification of TTV viral load and antibody response against the SARS-CoV-2 vaccine, TTV was detected in 76% of healthy participants, with a median of 2.9 log_10_ CP/mL, and in 97% of transplant recipients, with 3.9 log_10_ CP/mL. These results corroborate other findings in the literature, which demonstrate a higher TTV viral load in the transplant population^(42)^.

Finally, when relating the number of TTV viral replications (log_10_ CP/mL) and the clinical variables of the participants in this study, a moderate negative correlation was found between serum tacrolimus levels and the number of TTV viral copies (-0.463). This finding corroborates data from the literature that describe a decrease in TTV viral load with increasing doses of immunosuppressive drugs and an increase in TTV viral replication with decreasing immunosuppressive doses^(35,43,44)^. It has thus been demonstrated that quantifying TTV can be a strategy for adjusting the immunosuppressive dose and predicting infection and rejection in the transplant population.

### Study limitations

It is important to note a study limitation: participants were included at different times in the post-transplant period, with a maximum recruitment interval of 12 months after the procedure. The difficulties in recruiting participants stem from the fact that this is a highly complex procedure performed less frequently than transplantation in the adult population, which considerably reduces the available sample size. Additionally, as this is a novel comparison of transplantation and SARS-CoV-2 in the pediatric and adolescent renal transplant population, it was not possible to compare the findings with other literature on the same population.

### Contributions to nursing, health, or public policy

It is important to highlight the contributions of salivary analysis as an important viral detection technique, especially in this population, since it reduces invasive interventions and provides greater patient comfort during collection. Considering that viral infections and graft rejection are important unfavorable clinical situations for this population, efforts to use TTV as a predictor of these outcomes should be encouraged and implemented.

## CONCLUSIONS

This study identified salivary TTV excretion in kidney transplant recipients (children and adolescents) and correlated the number of TTV viral replications with SARS-CoV-2 PCR. The study of these variables in this population is noteworthy for its novelty.

Although the relationship between TTV and COVID-19 has not been explained by statistical methods, the findings suggest a trend towards increased TTV viral replication in the presence of SARS-CoV-2. Therefore, prospective studies are suggested to monitor TTV viral load and associated clinical outcomes (infection, rejection, and death), since this virus can be used as a biomarker of immunosuppression and severity.

Therefore, the results of this study reinforce the importance of monitoring TTV viral load in transplant patients, especially in childhood and adolescence. Furthermore, the use of saliva as a diagnostic biological fluid proved to be technically efficient, providing greater patient comfort during sample collection.

## Data Availability

The research data are available within the article.
