# Supplementary material for: Salivary Excretion Of Torque Teno Virus And Sars-Cov-2 In Children Who Underwent Kidney Transplantation
Source: Rev Bras Enferm. 2026 Sep 18;79:e20250285. doi: 10.1590/0034-7167-2025-0285 (PMC13588626; doi:10.1590/0034-7167-2025-0285)
Supplement: Translation

# Translation: Excreção Salivar De Torque Teno Vírus E Sars-Cov-2 Em Crianças Que Realizaram Transplante Renal

**DOI:** 10.1590/0034-7167-2025-0285pt

**Authors:** Ísis Oliveira Arruda, Richarlisson Borges de Morais, Rodrigo Melim Zerbinati, Karen Renata Nakamura Hiraki, Maria Cristina de Andrade, Dulce Aparecida Barbosa, Paulo Henrique Braz-Silva, Monica Taminato

**Affiliations:** I Universidade Federal de São Paulo. São Paulo, São Paulo, Brasil; II Universidade Federal de Uberlândia. Uberlândia, Minas Gerais, Brasil; III Universidade de São Paulo. São Paulo, São Paulo, Brasil

**Keywords:** Torque Teno Vírus, COVID-19, SARS-CoV-2, Transplante de Rim, Pediatria.

## Abstract

**Objetivos:**

verificar a excreção salivar de Torque Teno Vírus (TTV) em crianças e adolescentes transplantados renais e correlacionar o número de réplicas virais com o teste de reação em cadeia da polimerase para SARS-CoV-2.

**Métodos:**

estudo observacional transversal, realizado no ambulatório pós-transplante renal do Hospital do Rim e Hipertensão.

**Resultados:**

foram incluídos 31 pacientes, com predomínio do sexo masculino (54,8%) e idades entre 13 e 15 anos (38,7%). A excreção salivar de TTV foi detectada em 96,7% dos participantes, sendo que 36,7% também testaram positivo para SARS-CoV-2. No entanto, não foi encontrada associação significativa entre os dois vírus. Observou-se uma correlação moderada e negativa (-0,463) entre os níveis de TTV e a concentração sérica de tacrolimus.

**Conclusões:**

a excreção salivar de TTV foi identificada em crianças e adolescentes transplantados renais. Embora não tenha sido observada correlação entre TTV e SARS-CoV-2, houve associação entre TTV e níveis séricos de tacrolimus.

## INTRODUÇÃO

O Torque Teno Vírus (TTV), um anellovírus, foi encontrado pela primeira vez em 1997 no plasma sanguíneo de japoneses que desenvolveram hepatite após transfusão^(1-4)^. O TTV apresenta diversidade genética de mais de 29 espécies, com predominância de aproximadamente 95% na população mundial^(2,4)^, independentemente da idade, da situação econômica e das condições de saúde^(5-7)^.

O TTV é considerado um vírus órfão/comensal, pois, até o momento, a comunidade científica não conseguiu identificar sua associação a alguma doença^(8-10)^, sendo considerado parte do viroma humano em pessoas saudáveis. Alguns estudos relatam que a infecção primária do vírus ocorre na infância, levando à infecção crônica e a uma subsequente viremia estável^(11)^, porém sua forma de replicação e transmissão ainda é desconhecida^(12)^.

Nos últimos anos, as técnicas de biologia molecular permitiram descobrir que o TTV constitui mais de 65% do viroma em pacientes pós-transplante e apenas 10% em indivíduos imunocompetentes^(4,13,14)^. Os níveis plasmáticos de DNA do TTV mostraram oscilações em pacientes imunossuprimidos, o que significa que altas cargas virais de TTV têm associação direta com a intensidade da funcionalidade imunológica do indivíduo^(15-18)^. Assim, distúrbios imunológicos, medicamentos anti-inflamatórios ou imunossupressores podem afetar o equilíbrio imunológico e, consequentemente, favorecer a replicação do TTV^(8,11,19)^. Isso permite a identificação de altos níveis de carga viral do TTV e sua detecção em diferentes amostras, como plasma, saliva e soro^(8,17)^.

Nesse contexto, considerando que a Doença do Coronavírus 2019 (COVID-19) tem sido diretamente relacionada à desregulação inflamatória e imunológica, essas alterações estão associadas à gravidade do quadro clínico da COVID-19^(20)^. Dessa forma, tornou-se necessário compreender a interação do SARS-CoV-2 com outros patógenos e a influência dessas interações e de outras condições clínicas subjacentes no quadro clínico da doença.

Pacientes receptores de transplante renal apresentam um risco elevado para COVID-19 grave, devido ao seu estado de imunossupressão^(21)^. Além disso, estudos demonstram que pacientes imunossuprimidos, inclusive os transplantados renais, possuem maior dispersão de TTV quando comparados aos seus pares saudáveis^(22)^. Considerando esse risco aumentado, associado à coinfecção por SARS-CoV-2, há relatos na literatura que indicam mudanças no perfil de excreção viral para o TTV^(21)^. Diante do exposto, destaca-se que o paciente transplantado renal possui dupla associação de fatores que alteram o seu estado imunológico: a doença renal crônica (DRC) e o uso de medicamentos imunossupressores^(23)^. Essas características clínicas e epidemiológicas motivaram a realização do presente estudo, a fim de sanar a lacuna existente na literatura em relação à associação entre o número de réplicas virais e TTV e a coinfecção de SARS-CoV-2.

## OBJETIVOS

Verificar a excreção salivar de TTV em crianças e adolescentes transplantados renais e correlacionar o número de réplicas virais com o teste de reação em cadeia da polimerase (PCR) para SARS-CoV-2.

## MÉTODOS

### Aspectos éticos

O estudo foi conduzido conforme diretrizes éticas nacionais e internacionais. Foi aprovado pelo Comitê de Ética em Pesquisa da Universidade Federal de São Paulo (UNIFESP), cujo parecer está anexado à presente submissão.

### Tipo de estudo

Trata-se de estudo observacional do tipo transversal, em acordo com as recomendações das diretrizes *STrengthening the Reporting of OBservational studies in Epidemiology* ^(14)^ para estudos observacionais em epidemiologia^(24)^.

### Período e local do estudo

O estudo foi realizado no ambulatório de pós-transplante renal do Hospital do Rim e Hipertensão (HRim), da Fundação Oswaldo Ramos e da UNIFESP.

As coletas de material biológico (saliva) e dos dados sociodemográficos e clínicos dos prontuários eletrônicos foram realizadas no período de 30 dias, de 27 de janeiro a 26 de fevereiro de 2022, de todas as crianças e adolescentes que estavam em acompanhamento ambulatorial pós-transplante renal, na instituição participante.

### População do estudo

Trata-se de amostra por conveniência, composta por 31 indivíduos de 0 a 18 anos, com DRC terminal, em acompanhamento ambulatorial no HRim, submetidos ao transplante renal.

### Critérios de inclusão e exclusão

Foram incluídos no estudo pacientes pediátricos com idade igual ou inferior a 18 anos, que realizaram o transplante renal nos 12 meses anteriores ao período de coleta, em acompanhamento no ambulatório de pós-transplante do HRim.

### Protocolo do estudo

Os pais ou responsáveis pelos indivíduos elegíveis para a pesquisa foram abordados na sala de espera. Após a explanação e a compreensão dos objetivos e de todas as etapas da pesquisa, eles foram convidados a participar e a assinar o Termo de Consentimento Livre e Esclarecido (TCLE). Com a assinatura do TCLE, foram explicados ao participante (criança e/ou adolescente), em linguagem adequada, os objetivos do estudo e os procedimentos para a coleta da amostra de saliva antes de obter o seu assentimento para participação. Foi coletada uma amostra de saliva do participante em terapia renal substitutiva (TRS). Os dados relacionados às características sociodemográficas, ao histórico e à evolução clínica foram obtidos nos prontuários, utilizando-se um instrumento semiestruturado elaborado pelos pesquisadores a partir da revisão da literatura. Destaca-se que o estudo não gerou nenhuma mudança em sua rotina assistencial ou alteração no tratamento.

As amostras de saliva foram obtidas utilizando o método padronizado do dispositivo comercial Salivette^®^ (Sarstedt, Alemanha). Os tubos foram identificados e armazenados em refrigerador (entre 2 °C e 8 °C) até o fim das coletas previstas para o turno. Posteriormente, as amostras foram centrifugadas a 1.000 x g por dois minutos à temperatura de 20 °C, depois foram aliquotadas e congeladas em *freezer* a -80 °C.

O ácido nucleico (DNA/RNA) foi extraído a partir de 200 µL de cada amostra salivar com o uso do *kit* de extração de RNA e DNA viral (*Loccus* do Brasil Ltda.) no equipamento Extrator e Purificador de DNA e RNA - EXTRACTA 32. Para a detecção do RNA viral do SARS-CoV-2, foi realizada uma análise molecular com o uso do *kit* de reagentes para PCR quantitativa em tempo real com transcrição reversa do SARS-CoV-2 (PerkinElmer, Finlândia), seguindo as instruções do fabricante. A quantificação de réplicas virais de TTV foi realizada por meio de PCR quantitativo no equipamento *QuantStudio™ 6 Flex Real-Time PCR System* (*Applied Biosystems* ^®^, *Thermo Fisher*, EUA), com 45 ciclos de amplificação, conforme descrito previamente na literatura^(25)^.

### Análise estatística

A análise de dados foi realizada por meio de estatística descritiva, com medidas de frequência, de tendência central (média e mediana) e de dispersão (desvio padrão). Além disso, foram realizadas análises de comparação entre os grupos de acordo com a natureza das variáveis.

Ao comparar as variáveis numéricas, utilizou-se o teste não paramétrico de Mann-Whitney, e para as variáveis categóricas, utilizou-se o teste exato de Fisher. Para fazer a correlação entre o número de réplicas de TTV e as demais variáveis, utilizaram-se a correlação de Pearson, quando as variáveis apresentaram distribuição normal, e a correlação de Spearman, para aquelas que não possuíam distribuição normal. O nível de significância adotado foi o de 0,05, equivalendo a uma confiança de 95%.

## RESULTADOS

Foram incluídas 31 crianças e adolescentes transplantadas renais, que atenderam aos critérios de elegibilidade do presente estudo. Ao observar os dados sociodemográficos dos participantes, destacou-se a prevalência do sexo masculino (17/54,8%), faixa etária de 13 a 15 anos, (12/38,7%) etnia parda (16/51,6%), ensino fundamental incompleto (18/58,1%), renda familiar de até um salário mínimo (19/61,3%) e não beneficiários do Instituto Nacional do Seguro Social (18/58,1%). Quanto ao meio de transporte (19/61,3%), relataram utilizar carro para ir ao ambulatório pós-transplante renal, com tempo médio de trajeto superior a três horas (17/54,8%) (Tabela 1).

**Tabela 1:** Análise descritiva das características sociodemográficas, clínicas e laboratoriais de crianças e adolescentes transplantados renais, São Paulo, São Paulo, Brasil, 2024 (N=31)

CARACTERÍSTICAS	n (%)
**Sexo**	
Masculino	17 (54,8%)
Feminino	14 (45,2%)
**Idade**	
5 a 8 anos	4 (12,9%)
9 a 12 anos	7 (22,6%)
13 a 15 anos	12 (38,7%)
16 a 18 anos	8 (25,8%)
**Cor da pele/etnia**	
Branca	8 (25,8%)
Indígena	1 (3,2%)
Negra	6 (19,4%)
Parda	16 (51,6%)
**Escolaridade**	
Pré-escola	1 (3,2%)
Fundamental incompleto	18 (58,1%)
Ensino médio incompleto	11 (35,5%)
Não frequenta escola regular	1 (3,2%)
**Renda familiar (salário mínimo)**	
Até 1	19 (61,3%)
1-3	11 (35,5%)
**DADOS SOCIODEMOGRÁFICOS**	
**Recebe benefício do INSS**	
Sim	13 (41,9%)
Não	18 (58,1%)
**Meio de transporte utilizado para ir ao ambulatório pós-TX**	
Carro	19 (61,3%)
Ônibus	12 (38,7%)
Trem	3 (9,7%)
Metrô	9 (29%
Avião	4 (12,9%)
**Tempo de deslocamento (residência-ambulatório pós-TX)**	
Até uma hora	8 (25,8%)
De uma hora a duas horas	3 (9,7%)
De duas horas a três horas	3 (9,7%)
Mais de três horas	17 (54,8%)
**Situação da residência**	
Própria	17 (54,8%)
Alugada	8 (25,8%)
Cedida	6 (19,4%)
**Característica da residência**	
Alvenaria	31 (100%)
**Número de cômodos da residência**	
0	1 (3,2%)
2	3 (9,7%)
3	2 (6,5%)
4	10 (32,3%)
5	10 (32,3%)
6	4 (12,9%)
9	1 (3,2%)
**Número de residentes no mesmo domicílio**	
1	4 (12,9%)
3	9 (29%)
4	8 (25,8%)
5	5 (16,1%)
6	4 (12,9%)
8	1 (3,2%)
**Água encanada**	
Sim	28 (90,3%)
Não	3 (9,7%)
**Rede de esgoto**	
Sim	26 (83,9%)
Não	5 (16,1%)
**DADOS CLÍNICOS**	
**Etiologia da DCR**	
Indeterminada	9 (29%)
Síndrome hemofagocítica	1 (3,2%)
Síndrome nefrótica	6 (19,4%)
Secundária à lesão renal aguda	1 (3,2%)
Anomalias congênitas do rim e do trato urinário	6 (19,4%)
Nefropatia por imunoglobulina A	1(3,2%)
Bexiga neurogênica	2 (6,5%)
Displasia renal	1 (3,2%)
Rim policístico	1 (3,2%)
Refluxo vesicoureteral bilateral	3 (9,7%)
Arnold Chiari tipo II	1 (3,2%)
Síndrome hemolítico-urêmica	1 (3,2%)
Doença renal policística autossômica recessiva	1 (3,2%)
Cistinose nefropática	1 (3,2%)
**Houve TRS anterior**	
Sim	28 (90,3%)
Não	3(9,7%)
**Qual TRS anterior**	
Hemodiálise	27 (87,1%)
Diálise peritoneal automatizada	1 (3,2%)
**Tipo sanguíneo**	
A	10 (32,3%)
B	2 (6,5%)
AB	1 (3,2%)
O	18 (58%)
**Fator RH**	
Positivo	28 (90,3)
Negativo	3 (9,7%)
**Recebeu transfusão de sangue**	
Sim	16 (51,6%)
Não	15 (48,4%)
**Número de transfusões nos últimos 12 meses**	
<5	15 (48,3%)
6 a 10	1 (3,2%)
**Tipo de doador**	
Falecido	30 (96,7%)
Vivo relacionado	1(3,3%)
**Vacinado contra influenza (2022)**	
Sim	17 (54,8%)
Não	14 (45,2%)
**Vacinado contra COVID-19**	
Sim	29 (93,5%)
Não	2 (6,5%)
**EXAMES LABORATORIAIS**	
**Urocultura pós-TX**	
Positivo	1 (3,2%)
Negativo	19 (61,3%)
**Germe isolado na urocultura**	
*Klebsiella pneumoniae*	1 (3,2%)
**Hemocultura pós-TX**	
Positivo	0 (0%)
Negativo	18 (58%)

*N - número absoluto; % - número relativo; INSS - Instituto Nacional do Seguro Social; DRC - doença renal crônica; TRS - terapia renal substitutiva; HD - hemodiálise; TX - transplante renal.*

Quanto às características residenciais, a maioria tem casa própria (17/54,8%), construída em alvenaria (31/100%), com quatro ou cinco cômodos (10/32,3%), e a maioria tem três residentes na mesma habitação (9/29%). A maioria tem água encanada (28/90,3%) e rede de esgoto (83,9%) (Tabela 1).

Em relação aos dados clínicos, a etiologia da DRC (9/29%) foi considerada indeterminada, sendo as mais prevalentes síndrome nefrótica e anomalias congênitas do rim e do trato urinário (6/19,4%). Ressalta-se que 28 (90,3%) passaram por outro método de TRS antes do transplante renal, sendo que 27 (87,1%) fizeram hemodiálise. O tipo sanguíneo mais prevalente foi o “O” (18/90,3%), com predominância do fator Rh positivo (28/90,3%). Os pacientes receberam transfusão em menos de cinco ocasiões (15/48,3%), foram submetidos ao transplante de doador falecido (30/96,7%) e receberam vacina contra influenza (17/54,8%) e contra a doença do coronavírus 2019 (29/93,5%). No que se refere aos exames laboratoriais, um paciente apresentou urocultura positiva (1/3,2%) para *Klebsiella pneumoniae*, e todas as hemoculturas coletadas tiveram resultado negativo.

De acordo com a Figura 1, entre os 31 participantes do estudo, 30 (96,8%) apresentaram dispersão de réplicas virais de TTV, enquanto em um (3,2%) não foram encontradas tais réplicas na saliva. Além disso, 11 participantes (35,5%) apresentaram resultado positivo para PCR salivar concomitante ao TTV. Destaca-se que, ao aplicar o teste exato de Fisher, não houve associação estatisticamente significante entre os vírus TTV e SARS-CoV-2 (p=1).

**Figura 1:** Resultado do teste de reação em cadeia da polimerase qualitativo para Torque Teno Vírus e SARS-CoV-2 de crianças e adolescentes transplantados renais, São Paulo, São Paulo, Brasil, 2024 (N=31)

Ao comparar as variáveis sociodemográficas, clínicas e laboratoriais das crianças e adolescentes transplantados renais incluídos no presente estudo, com o PCR qualitativo para TTV, nota-se que não houve associação estatisticamente significativa com nenhuma das variáveis estudadas.

Ao comparar o número de réplicas virais de TTV identificadas na saliva de crianças e adolescentes transplantados renais com o resultado qualitativo do PCR salivar para o SARS-CoV-2, não foi observada relação entre ter ou não ter a doença e o número de réplicas virais de TTV (p=0,426) (Tabela 2).

**Tabela 2:** Comparação entre o número de réplicas virais de Torque Teno Vírus e reação em cadeia da polimerase quantitativa em tempo real com transcrição reversa para SARS-CoV-2 em crianças e adolescentes transplantados renais, São Paulo, São Paulo, Brasil, 2024 (N=31)

	RT-PCR SARS-CoV- 2		
**TTV log_10_ CP/mL**	**Positivo**	**Negativo**	Valor de *p* ^^*^^
**Média (±)**	9,304 (1,656)	8,768 (1,925)	0,426

TTV - Torque Teno Vírus; RT-PCR - reação em cadeia da polimerase quantitativa em tempo real com transcrição reversa; log_10_ - logaritmo de base 10; CP/mL - cópias por mililitro;

* Teste de Mann-Whitney.

Na Tabela 3, tem-se a correlação entre o número de réplicas virais de TTV (log_10_ CP/mL) e as variáveis clínicas. Pode-se destacar que há uma correlação moderada e negativa entre tacrolimo sérico e TTV (log_10_ CP/mL). Para as demais variáveis quantitativas, a correlação com TTV log_10_ CP/mL se mostrou fraca.

**Tabela 3:** Correlação entre Torque Teno Vírus e variáveis sociodemográficas, clínicas e laboratoriais em crianças e adolescentes transplantados renais, São Paulo, São Paulo, Brasil, 2024 (N=31)

Correlação	TTV log_10_ CP/mL
Tempo deslocamento horas	-0,129^*^
Número de cômodos	0,181^*^
Residentes mesma casa	-0,083^*^
Idade	0,034^*^
Peso	0,105^**^
Altura	0,157^*^
Tempo de início da TRS	0,042 ^*^
Tempo de TX em anos	-0,294^**^
Tempo de TX em meses	-0,294^**^
Número de episódios de ITU pós-TX	0,237 ^*^
Número de episódios de ICS pós-TX	0,308^*^
Glicose pré-TX	-0,065^**^
Creatinina pré-TX	0,298^**^
Tempo de isquemia (em horas)	-0,369^*^
Tempo de isquemia fria (em horas)	-0,365^*^
Tempo de isquemia quente (em minutos)	0,267^*^
Tacrolimo sérico	-0,463^**^
Cr	-0,179^*^
K	-0,171^*^
Ht	-0,167^**^
Hb	-0,149^**^
Leu	-0,197^**^

TRS - terapia renal substitutiva; TX - transplante renal; ITU - infecção do trato urinário; ICS - infecção de corrente sanguínea; Cr - creatinina; K - potássio; Ht - hematócrito; Hb - hemoglobina; Leu - leucócitos; log10 - logaritmo de base 10; CP/mL - cópias por mililitro;

* Correlação de Spearman;

** Correlação de Pearson.

## DISCUSSÃO

O presente estudo verificou a excreção salivar do TTV em crianças e adolescentes submetidos ao transplante renal, avaliando a associação entre o número de réplicas virais de TTV e a detecção de SARS-CoV-2. Os resultados revelaram que 96,8% dos participantes apresentaram positividade para o TTV, com carga viral média de 9,304 log_10_. Nos indivíduos com coinfecção por SARS-CoV-2, houve uma discreta variação na carga viral do TTV (8,768 log_10_), sem diferença estatisticamente significativa.

A distribuição sociodemográfica dos participantes foi consistente com estudos anteriores, que indicam maior prevalência do TTV no sexo masculino^(23,26)^. Estudo em doadores de sangue italianos demonstrou uma prevalência de 65%, com aumento progressivo da detecção do vírus em indivíduos abaixo dos 60 anos, alcançando 45% entre 18 e 22 anos^(27)^. Outra investigação realizada com 313 indivíduos saudáveis relatou que 70% das amostras de plasma positivo para TTV eram de indivíduos entre 20 e 30 anos, com 78% na idade entre 50-60 anos e 80% na faixa etária de 80 anos. Além disso, nos participantes saudáveis, houve associação entre sexo e carga viral, sendo menores em mulheres jovens em comparação aos homens na mesma faixa etária^(28)^.

Em relação à interação entre TTV e COVID-19, estudo conduzido pelo “Programa Corona São Caetano”, no município de São Caetano do Sul, Brasil, revelou a presença do TTV em 54,6% dos indivíduos positivos para o SARS-CoV-2 e em 62,7% dos controles. O estudo também identificou uma redução significativa na carga viral do TTV após dez dias de evolução clínica da COVID-19, enquanto que, nos indivíduos negativos, a carga permaneceu inalterada^(29)^. Da mesma forma, relato de caso envolvendo um paciente renal transplantado demonstrou um aumento na carga de TTV durante a infecção por SARS-CoV-2 (7,14 log_10_/7,87 log_10_). Ao comparar esse achado com o número de réplicas virais de TTV nos meses um e seis pós-transplante (5,6 log_10_ e 5,9 log_10_, respectivamente), nota-se que houve um aumento da dispersão viral de TTV durante o quadro de infecção por SARS-CoV-2, o que sugere sua potencial utilidade como biomarcador de imunossupressão^(30)^.

Estudos indicam que a monitorização da carga viral do TTV pode ser um indicador confiável do estado imunológico em pacientes transplantados^(31)^. As evidências demonstram a relação entre a variação dos níveis de TTV e a função imunológica do paciente transplantado^(32-34)^.

O uso de medicamentos imunossupressores reduz o risco de rejeição do transplante, porém aumenta o risco de infecção^(35)^. Estudos evidenciam que quantificar a carga viral de TTV tem muitos benefícios, visto que o PCR pode ser feito em poucas horas, é disponível em muitas instituições de saúde, além de ser uma técnica já consolidada. Ademais, a carga viral de TTV pode predizer rejeição^(34,35)^ e infecção em pacientes transplantados, o que contribui para o acompanhamento e tomada de decisão clínica^(36)^.

Estudos prospectivos sugerem que a quantificação da carga viral do TTV após 180 dias do transplante renal pode auxiliar na calibração da terapia imunossupressora, reduzindo riscos de infecção e rejeição^(37,38)^. Pesquisadores propõem um intervalo ideal de carga viral entre 4,6 e 6,6 log_10_ CP/mL após 90 dias de transplante. Tal resultado indicou que valores inferiores a 4,6 log_10_ podem estar associados à rejeição, e valores superiores a 6,6 log_10_ podem aumentar o risco de infecção^(39)^.

Outro estudo austríaco com 386 adultos transplantados observou que 68 participantes rejeitaram o transplante e 207 apresentaram 472 episódios de infecção nos primeiros 12 meses. Após três meses do transplante, o estudo relatou que os níveis de corte para a faixa de TTV para rejeição foram a mediana de TTV (3,5 × 10^6^ c/mL) e a mediana para a ocorrência de infecção (3,9 × 10^8^ c/mL)^(40)^.

Com o surgimento da emergência de saúde pública relacionada à COVID-19, muitos estudos propuseram relacionar a infecção por SARS-CoV-2 em diferentes situações clínicas, incluindo a coinfecção por outros patógenos. Estudo comparou a mortalidade por COVID-19 em um centro de transplante renal no período de março de 2020 a dezembro de 2022 em relação aos cinco anos anteriores (2014/2019). Identificou-se que houve, no período antes da pandemia, 1.025 vidas ceifadas por ano, enquanto que na era pandêmica houve 1.429 óbitos, sendo a COVID-19 responsável por 59% das mortes^(20)^. A imunização contra a COVID-19 em pacientes com DRC é um aspecto crucial a ser considerado. Estudos enfatizam a segurança e a eficácia das vacinas em crianças com comorbidades, reforçando que os benefícios sobrepõem os riscos^(41)^.

Coorte retrospectiva realizada em Roma com 172 pacientes transplantados de órgãos sólidos (rim e pulmão) e 72 doadores de sangue, para correspondência cruzada com a população saudável, avaliou a relação entre a carga viral do TTV e a resposta humoral após a administração das doses da vacina de RNA mensageiro contra a COVID-19, BNT162B2. Constatou-se que 52% dos pacientes apresentaram anticorpos após a segunda dose da vacina, 76% após a terceira dose e 87% após a quarta dose. Quanto à quantificação da carga viral do TTV e à resposta de anticorpos contra a vacina de SARS-CoV-2, o TTV foi detectado em 76% dos participantes saudáveis, com mediana de 2,9 log_10_ CP/mL, e em 97% dos transplantados, com 3,9 log_10_ CP/mL. Esses resultados corroboram outros achados da literatura, que demonstram uma carga viral maior de TTV na população transplantada^(42)^.

Por fim, ao relacionar o número de réplicas virais de TTV (log_10_ CP/mL) e as variáveis clínicas dos participantes do presente estudo, evidencia-se correlação moderada e negativa entre a dosagem sérica de tacrolimo e o número de cópias virais de TTV (-0,463). Esse achado corrobora dados da literatura que descrevem a diminuição da carga viral de TTV com o aumento da dose de medicamentos imunossupressores e o aumento da replicação viral de TTV com a diminuição da dose imunossupressora^(35,43,44)^. Demonstrou-se, assim, que quantificar o TTV pode ser uma estratégia para ajustar a dose imunossupressora e predizer infecção e rejeição na população transplantada.

### Limitações do estudo

Como limitação do presente estudo, é importante destacar que os participantes foram incluídos em diferentes momentos do período pós-TX, respeitando o intervalo de, no máximo, 12 meses após o procedimento para recrutamento. Destaca-se que as dificuldades de recrutamento de participantes se dão pelo fato de se tratar de um procedimento de alta complexidade, executado em menor frequência quando comparado com o transplante na população adulta, o que diminui consideravelmente a amostra disponível. Além disso, por se tratar de uma comparação inédita (TTV e SARS-CoV-2) para a população pediátrica e hebiátrica transplantada renal, não foi possível comparar os achados com outras evidências da literatura para a mesma população.

### Contribuições para as áreas da enfermagem, saúde ou políticas públicas

Cabe destacar a análise salivar, importante técnica de detecção viral, especialmente nesta população, pois diminui as intervenções invasivas e proporciona maior conforto ao paciente durante a coleta. Considerando que as infecções virais e a rejeição do enxerto são importantes situações clínicas desfavoráveis para esta população, esforços para a utilização do TTV como preditor desses desfechos devem ser estimulados e implementados.

## CONCLUSÕES

Este estudo identificou a excreção salivar de TTV em crianças e adolescentes transplantados renais e relacionou o número de réplicas virais de TTV com o PCR para SARS-CoV-2. Destaca-se o ineditismo do estudo dessas variáveis para esta população.

Apesar de a relação entre TTV e COVID-19 não ter sido explicada pelos métodos estatísticos, os achados sugerem a tendência no aumento de réplicas virais de TTV na presença do SARS-CoV-2. Diante disso, sugere-se a realização de estudos prospectivos, a fim de acompanhar a carga viral de TTV e os desfechos clínicos (infecção, rejeição e óbito) a ela associados, visto que esse vírus pode ser utilizado como um biomarcador de imunossupressão e de severidade.

Sendo assim, os resultados do presente estudo reforçam a importância da monitorização da carga viral do TTV em pacientes transplantados, especialmente na infância e adolescência. Além disso, o uso da saliva como fluido biológico diagnóstico se mostrou eficiente do ponto de vista técnico, proporcionando maior conforto ao paciente no momento da coleta da amostra.

## Data Availability

Os dados de pesquisa estão disponíveis no corpo do artigo.
